# Behavior Change Techniques in Wrist-Worn Wearables to Promote Physical Activity: Content Analysis

**DOI:** 10.2196/20820

**Published:** 2020-11-19

**Authors:** Peter Düking, Marie Tafler, Birgit Wallmann-Sperlich, Billy Sperlich, Sonja Kleih

**Affiliations:** 1 Integrative and Experimental Exercise Science Department of Sport Science Würzburg Germany; 2 Department of Psychology I University of Würzburg Würzburg Germany; 3 Institute for Sport Sciences University of Würzburg Würzburg Germany

**Keywords:** cardiorespiratory fitness, innovation, smartwatch, technology, wearable, eHealth, mHealth

## Abstract

**Background:**

Decreasing levels of physical activity (PA) increase the incidences of noncommunicable diseases, obesity, and mortality. To counteract these developments, interventions aiming to increase PA are urgently needed. Mobile health (mHealth) solutions such as wearable sensors (wearables) may assist with an improvement in PA.

**Objective:**

The aim of this study is to examine which behavior change techniques (BCTs) are incorporated in currently available commercial high-end wearables that target users’ PA behavior.

**Methods:**

The BCTs incorporated in 5 different high-end wearables (Apple Watch Series 3, Garmin Vívoactive 3, Fitbit Versa, Xiaomi Amazfit Stratos 2, and Polar M600) were assessed by 2 researchers using the BCT Taxonomy version 1 (BCTTv1). Effectiveness of the incorporated BCTs in promoting PA behavior was assessed by a content analysis of the existing literature.

**Results:**

The most common BCTs were goal setting (behavior), action planning, review behavior goal(s), discrepancy between current behavior and goal, feedback on behavior, self-monitoring of behavior, and biofeedback. Fitbit Versa, Garmin Vívoactive 3, Apple Watch Series 3, Polar M600, and Xiaomi Amazfit Stratos 2 incorporated 17, 16, 12, 11, and 11 BCTs, respectively, which are proven to effectively promote PA.

**Conclusions:**

Wearables employ different numbers and combinations of BCTs, which might impact their effectiveness in improving PA. To promote PA by employing wearables, we encourage researchers to develop a taxonomy specifically designed to assess BCTs incorporated in wearables. We also encourage manufacturers to customize BCTs based on the targeted populations.

## Introduction

Various forms of physical activity (PA) reduce the incidence of noncommunicable diseases, obesity, and mortality. However, according to the World Health Organization (WHO), levels of physical inactivity are increasing with approximately 28% of adults failing to meet PA guidelines [1]. Therefore, measures to increase PA are urgently needed.

Behavioral PA interventions (eg, employing cognitive and behavioral techniques to modify and increase PA behavior) successfully increase PA [2]. However, these interventions target smaller groups of mostly previously motivated participants. The eHealth and mobile health (mHealth) [3] solutions employing wearable sensors (wearables) may encourage various populations to increase their levels of PA on a larger scale. Wearables monitor certain components of PA via surrogate markers (eg, movement acceleration converted to metabolic equivalents or electrical signals converted to heart rate) [4] and provide biofeedback [5], thereby potentially assisting in elevating PA. A recent review concludes that wearables have the potential to increase PA participation as long as wearables are the primary component of an intervention or part of a broader intervention [6]. Additionally, the WHO aims to endorse digital health concepts [7]. In Germany, physicians are permitted to prescribe digital health solutions if proven to be effective [8]. At the same time, wearable-assisted interventions may be more cost-effective than traditional interventions [9].

To promote PA, different behavior change techniques (BCT) can be incorporated in wearables, which likely have different outcomes for promoting PA [3]. The selection of wearables using appropriate BCTs based on specific research questions and goals of healthy behavior is particularly crucial in the continuously growing wearable market. Manufacturers are releasing new models with rapidly changing features at least once every year, creating more choices for researchers and consumers.

Currently, little is known about how wearables differ from each other and which technologies are more effective in increasing levels of PA. Therefore, this study aimed to examine which BCTs targeting PA behavior are incorporated in the commercially available high-end wearables.

## Methods

### Wearables

For our analysis, we chose wearables manufactured by leading companies in the market [10]. The 5 wrist-worn wearables were the Apple Watch Series 3 (Apple Inc), Fitbit Versa (Fitbit Inc), Garmin Vívoactive 3 (Garmin), Polar M600 (Polar Electro Oy), and Xiaomi Amazfit Stratos 2 (Huami Technology). Each wearable was installed as instructed by the respective manufacturer and was synchronized with the companion app for smartphones (Apple iPhones).

### Coding Procedure

Wearables and companion apps were coded using the BCT Taxonomy Version 1 (BCTTv1), which was previously employed in similar studies [11-13]. The BCTTv1 is explained in detail by Michie et al [14]. Briefly, the BCTTv1 incorporated 93 nonredundant techniques, grouped into 16 hierarchical clusters in total, each coded using a dichotomous score of either 0 or 1, indicating nonpresence or presence, respectively [14].

Each wearable was worn by 2 researchers (MT and PD) for 1 week. The 2 researchers were well acquainted with the handling of the wearables, using the companion apps, and employing the BCTTv1. The researchers completed the training on the BCTTv1 website [15] before the analysis. Interrater reliability assessing each wearable incorporating a BCT was calculated using a kappa statistic in SPSS 22.0 (IBM Corp). Magnitude of agreement was interpreted as per the following criteria: 0.00=poor, 0.01-0.20=slight, 0.21-0.40=fair, 0.41-0.60=moderate, 0.61-0.80=substantial, and 0.81-1.00=almost perfect [16].

Coding disagreements were resolved by a discussion between the researchers. In case of disagreement, a third researcher's opinion (SK) was included to resolve the disagreement.

In line with the aim of this study, BCTs targeting PA were examined, while the feedback on other factors (eg, sleep or diet) was ignored. The researchers were instructed to include periods of physical inactivity as well as those of PA into the assessment week to verify the corresponding feedback by wearables.

### Evaluating the Effectiveness of BCTs in the Wearables to Promote PA

To evaluate the potential effects of incorporated BCTs, we employed a previous list [9] created for the same purpose. The list is based on a meta-analysis [17,18], meta-regression [19], and systematic reviews [20-22] as well as recommendations from the US Preventive Services Taskforce [23]. These BCTs were marked with checkmarks in Table 1. As in the earlier study [9], we used this list to count the number of effective BCTs, which were incorporated in each wearable to promote PA.

## Results

Table 1 summarizes the different BCTs incorporated in the 5 different wearables. Techniques from the taxonomy not immanent in any of the systems were excluded from the table. One disagreement between the original 2 researchers was solved by the opinion of the third one. The interrater reliability was almost perfect (Cohen kappa=0.965).

**Table 1:** Behavior change techniques incorporated in different wrist-worn wearables.

BCTs^a^	Proven effectiveness to promote physical activity	Apple Watch Series 3	Fitbit Versa	Garmin Vívoactive 3	Polar M600	Xiaomi Amazfit Stratos 2	Incorporations, N
Goal setting (behavior) (item 1.1)	✓	✓	✓	✓	✓	✓	5
Barrier identification/problem solving (item 1.2)	✓						0
Action planning (item 1.4)	✓	✓	✓	✓	✓	✓	5
Review behavior goal(s) (item 1.5)	✓	✓	✓	✓	✓	✓	5
Discrepancy between current behavior and goal (item 1.6)		✓	✓	✓	✓	✓	5
Commitment (item 1.9)	✓						0
Feedback on behavior (item 2.2)	✓	✓	✓	✓	✓	✓	5
Self-monitoring of behavior (item 2.3)	✓	✓	✓	✓	✓	✓	5
Biofeedback (item 2.6)	✓	✓	✓	✓	✓	✓	5
Social support (unspecified) (item 3.1)	✓	✓	✓	✓	✓	✓	5
Social support (emotional) (item 3.3)			✓	✓	✓		3
Instruction on how to perform the behavior (item 4.1)	✓		✓^b^	✓			1 (2)
Information about health consequences (item 5.1)	✓		✓	✓	✓	✓	4
Information about social and environmental consequences (item 5.3)	✓						0
Monitoring of emotional consequences (item 5.4)					✓		1
Information about emotional consequences (item 5.6)	✓						0
Demonstration of the behavior (item 6.1)	✓		✓^b^	✓			1 (2)
Social comparison (item 6.2)	✓	✓	✓	✓	✓	✓	5
Prompts/cues (item 7.1)	✓	✓	✓	✓	✓	✓	5
Behavioral practice/rehearsal (item 8.1)	✓		✓^b^	✓			1 (2)
Graded tasks (item 8.7)	✓	✓	✓	✓			2
Credible source (item 9.1)			✓	✓		✓	3
Nonspecific reward (item 10.3)	✓	✓	✓	✓	✓		4
Social reward (item 10.4)		✓	✓	✓	✓	✓	5
Nonspecific incentive (item 10.6)		✓	✓	✓			3
Self-reward (item 10.9)	✓						—^c^
Adding objects to the environment (item 12.5)		✓	✓	✓	✓	✓	5
Reward approximation (item 14.4)	✓		✓				1
Situation-specific reward (item 14.6)		✓	✓	✓			3
Verbal persuasion about capability (item 15.1)		✓		✓			2
Focus on past success (item 15.3)	✓	✓	✓	✓		✓	4
Self-talk (item 15.4)	✓						—
BCT clusters, n		10	13	13	8	10	—
Incorporated BCTs, n		18	24	24	16	15	—
Incorporated BCTs with proven effectiveness [17-23], n		12	17	16	11	11	—

^a^BCT: behavior change technique.

^b^Available as a paid add-on feature.

^c^Not applicable.

### Incorporated BCTs

Out of the 93 BCTs analyzed by the BCTTv1, 26 different BCTs were incorporated in the 5 wearables. On average, 19 BCTs (range 15-24) were incorporated in the wearables. Fitbit Versa and Garmin Vívoactive 3 incorporated the most BCTs (n=24), followed by Apple Watch Series 3 (n=18), Polar M600 (n=16), and Xiaomi Amazfit Stratos 2 (n=15). Due to technical issues with the Xiaomi Amazfit Stratos 2 device, we could not evaluate the BCT item of social support (emotional) (item 3.3); thereby, that item was not marked as incorporated with a checkmark in Table 1. Multimedia Appendix 1 provides detailed information about how often a BCT was incorporated in each wearable.

According to Lyons et al [9], 23 BCTs are effective in promoting PA. Out of these BCTs, Garmin Vívoactive 3 (n=16) and Fitbit Versa (n=14 + 3 paid BCTs) incorporate most BCTs, followed by Apple Watch Series 3 (n=12), Polar M600 (n=11), and Xiaomi Amazfit Stratos 2 (n=11). Only Fitbit Versa and Garmin Vívoactive 3 included the BCT items of instruction on how to perform behavior and behavioral practice/rehearsal. None of the wearables included the following BCT items: barrier identification/problem solving, self-reward, self-talk, commitment, information about social and environmental consequences, and information about emotional consequences.

### Clusters

On average, 11 (range 8-13) clusters were incorporated in the 5 wrist-worn wearables. Fitbit Versa and Garmin Vívoactive 3 incorporated most clusters (n=13), followed by Apple Watch Series 3 (n=10), Amazfit Stratos 2 (n=10), and Polar M600 (n=8). The 3 most common clusters were goals and planning (cluster 1) (n=4), feedback and monitoring (cluster 2) (n=3), and reward and threat (cluster 10) (n=3).

## Discussion

This study was designed to examine which BCTs were incorporated in the leading high-end wearables to promote PA. Our major findings were as follows:

1. The most common BCTs were goal setting (behavior), action planning, review behavior goal(s), discrepancy between current behavior and goal, feedback on behavior, self-monitoring of behavior, biofeedback, social support (unspecified), social comparison, prompts/cues, social reward, and adding objects to the environment.
2. Wearables often incorporate the same BCTs according to the BCTTv1. However, Fitbit Versa and Garmin Vívoactive 3 provided the most and Xiaomi Amazfit Stratos 2 provided the least number of BCTs.
3. Fitbit Versa (n=17) and Garmin Vívoactive 3 (n=16) offered the most BCTs, which showed to be effective to promote PA, while Xiaomi Amazfit Stratos 2 and Polar M600 had the least number of BCTs (n=11).

The number of incorporated BCTs in this study is in line with previous research examining incorporated BCTs within earlier versions of the wearables tested herein [11-13]. In studies comparing different wearables, Fitbit incorporated a higher number of BCTs than those incorporated by the Garmin [12] or Polar [11,13] wearables.

Absolute numbers of BCTs incorporated in wearables might not effectively inform about which wearables seem suitable to increase levels of PA. People’s decision making can be deteriorated by information overload and may even result in negatively perceived stress [24,25]. Consequently, everyone requires an optimal level of information quantity. In this regard, fewer but more effective BCTs may be preferable to promote PA [9].

A combination of BCTs (with proven effectiveness) may maximize effectiveness in promoting PA. A meta-analysis revealed that (1) “provide information about behavior–health link” combined with “prompt intention formation” (mean effect size, *g*=0.46) and (2) “provide information about behavior–health link” combined with “provide information on consequences” and “use of follow-up prompts” (mean effect size, *g*=0.44) were the most successful BCTs to alter health behavior [26]. When converted to the BCTTv1 taxonomy, the equivalent BCTs are (1) information about health consequences (item 5.1) combined with action planning (item 1.4), and (2) information about health consequences (item 5.1) with prompts/cues (item 7.1), respectively. A total of 4 devices tested herein (those manufactured by Fitbit, Garmin, Polar, and Xiaomi) incorporated all these BCTs; while the device manufactured by Apple incorporated all the BCTs except the information about health consequences (item 5.1).

Although the aforementioned BCTs and their combinations are effective in promoting PA on group level, effective BCTs may differ for specific populations and on an individual level. In fact, individual characteristics (eg, age, PA level, and personality) play a key role in a person’s choices and continuous device usages [27]. Consequently, it may be worthwhile to customize the incorporated BCTs in wearables to meet individual characteristics for PA promotion.

On an individual level, the effectiveness of BCTs and the choice of a wearable depend on practical factors. For example, BCTs incorporated in wearables are only effective when worn, which (among other factors) depend largely on personal preferences, including design and texture, battery life, and handling [27]. Additionally, sensor data that prompt different BCTs need reliable information [28]. For example, if a wearable uses inertial measurement units and optical heart rate sensors to calculate a person’s energy expenditure (a surrogate marker of PA) for prompting different BCTs, the sensor data and algorithms need to be as accurate as possible. Otherwise, the BCTs may mislead and ultimately fail personal PA goals. In our experience, companies often do not disclose the information of sensor data and algorithms applied to prompt a certain BCT. Transparency in this regard would assist in understanding the prompting of BCT and identifying preferred wearables to promote PA.

An effective increase of PA also depends on the user’s awareness of PA and its impact on health [29]. Using wearables without such knowledge might not represent an optimal option to improve PA. Although a recent meta-analysis showed that wearables improve PA (ie, measured by the number of daily steps, moderate to vigorous PA, and energy expenditure) discretely, a combination of wearables with other BCTs (eg, telephone counseling or group-based education) shows better results in increasing levels of PA [6].

Since wearables are commercially available, they can be used by consumers without consulting an expert opinion (eg, opinion of an exercise physiologist or a health expert). Potentially, the lack of professional guidance can induce unfavorable or dangerous PA participation (eg, by increasing PA too rapidly).

### Limitations

Although the taxonomy we applied to assess BCTs in wearables was employed previously [9], this taxonomy was not directly developed for machine to person (ie, wearable to consumer) interaction but for person to person (ie, psychologist to patient) interaction. This modification results in some difficulties in the application of this taxonomy. For example, different surrogate markers of PA (eg, number of steps or energy expenditure) can be employed to prompt the same BCT. To the best of our knowledge, it remains unclear which surrogate marker is most suitable as a basis for BCTs to improve PA.

Additionally, the BCTTv1 taxonomy does not allow certain functions, for example, evaluating how a BCT is visualized, evaluating how frequently BCTs are implemented (eg, daily report and hourly feedback), and determining whether a BCT is interactive (eg, a BCT disappears from the screen when an increase in PA is detected). However, we assume that these aspects affect the effectiveness of the BCTs and hence, that of the respective wearables in altering PA behavior among users.

Since the number of wearables promoting PA is rapidly increasing, we urge researchers to develop a taxonomy covering these aspects to assess the preferred BCTs employed by wearables.

Some BCTs displayed by the wearables addressed sedentary behavior as well as PA (ie, prompts/cues). Sedentary behavior is recognized as an independent behavior of PA [30,31]. In this study, we did not separate BCTs in terms of PA behavior and sedentary behavior. However, sedentary behavior and PA follow unique intervention logic [31,32], and research suggests that different factors influence each behavior. Future research should analyze the incorporated BCTs in wearables for sedentary behavior, separately of PA. Targeting both behaviors can have greater health benefits.

The shelf life of consumer-grade wearables, such as that of the devices we analyzed in this study, is short. Manufacturers frequently release new models, while older ones disappear from the market. Consequently, frequent assessment of new wearables, ideally before the market release (as recommended elsewhere [28]) is warranted.

### Conclusions

Based on our analysis, we conclude that out of all the tested wearables, Fitbit Versa (n=14+3 paid) and Garmin Vívoactive 3 (n=16) included most of the BCTs with proved effectiveness, followed by Apple Watch 5 (n=12), Polar M600 (n=11), and Xiaomi Amazfit Stratos 2 (n=11). Out of all the tested wearables, Garmin Vívoactive 3 and Fitbit Versa might be the most promising wearables to promote PA from a psychological perspective. However, future studies need to evaluate the effectiveness of these devices in experimental studies along with the validity and reliability of variables obtained by these devices. Since no specific taxonomy is available to investigate BCTs incorporated in digital health tools, including wearables for promoting PA, we advise developing such a taxonomy given the global urgency to improve PA and the increasing popularity and availability of wearables. Since the effectiveness of BCTs is affected by individual characteristics, we recommend manufacturers to allow customization of BCTs.

## Appendix

Multimedia Appendix 1 Examples of the behavior change techniques incorporated in the wearables tested herein.

## Abbreviations

BCT: behavior change technique
BCTTv1: BCT Taxonomy version 1
mHealth: mobile health
PA: physical activity
WHO: World Health Organization
