# Supplementary material for: Behavior Change Techniques in Wrist-Worn Wearables to Promote Physical Activity: Content Analysis
Source: JMIR Mhealth Uhealth. 2020 Nov 19;8(11):e20820. doi: 10.2196/20820 (PMC7714647; doi:10.2196/20820)
Supplement: Multimedia Appendix 1 [file mhealth_v8i11e20820_app1.pdf]

BCTs	Fitbit	Garmin	Polar	Xiaomi	Apple
1.1. Goal setting (behavior)	App - Goal setting for daily activity including steps, distance and burned calories. 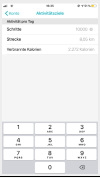	App - Goal setting for daily activity measured in steps per day with the opportunity to choose a preset goal based on previous activity. 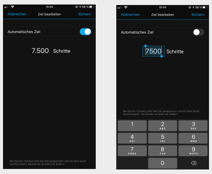	App - Selection from three preset activity goals differing in their intensities. 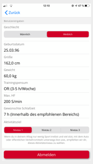	App - Goal setting for daily activity measured in steps per day. 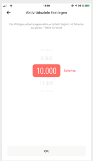	Wearable, App - Goal setting for daily activity measured in calories per day by the user or selection from three preset caloric goals depending on the user's personal activity goals. 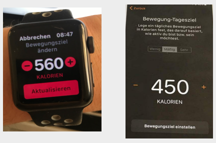
1.4. Action planning	App - Planning of daily physical activity, including steps, calories, distance, climbed floors per day as well as 250 steps per hour. 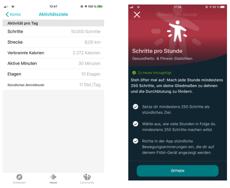	App - Planning of daily physical activity, including steps and climbed floors per day as well as minutes of intensity physical activity. 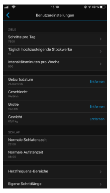	Web-App - Planning of daily physical activity by choosing one of three preset intensitiy levels. 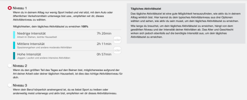	App - Planning of daily physical activity by setting a step goal. 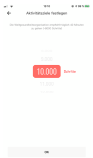	Wearable - Planning of daily physical activity by setting a calorie goal. 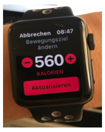
1.5. Review behavior goal(s)	App - Opportunity to change the previously set activity goals. 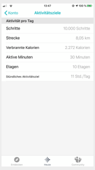	App - Opportunity to change the previously set activity goals. 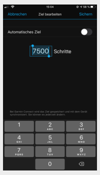	App, Web-App - Opportunity to change the previously set activity goals. 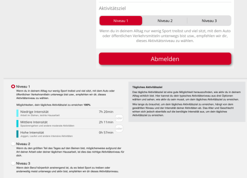	App - Opportunity to change the previously set activity goals. 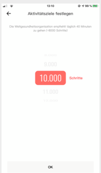	Wearable - Opportunity to change the previously set activity goals. 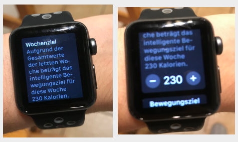
1.6. Discrepancy between current behavior and goal	Wearable - An indication that 126 steps are still missing to achieve the hourly movement goal of 250 steps. 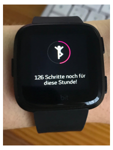	Wearable - Promt that at this time of day, 0 out of 10,100 steps, the daily activity goal, had been completed. 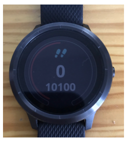	Wearable, App - The picture on the left informs the user that half of the daily activity goal has been reached, the right picture shows that 92% of the daily activity goal has been reached. 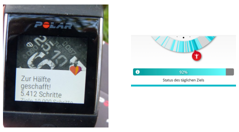	App - Information that 4618 steps have already been completed and 5382 are still necessary to achieve the daily step goal. 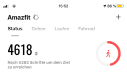	Wearable - Information about the daily progress in reaching the activity goals. In this case, 14 of the 560 calories required have already been burned.In addition, 0 of the required 30 minutes of physical training was achieved and one of the required 12 hours was spent standing. 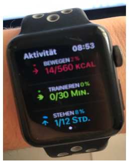
2.2. Feedback on behaviour	App - Daily feedback on calories burned, steps taken, floors climbed, distance traveled and number of minutes spent on physical activity 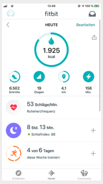	App - Feedback on the steps taken in the past few months including the total number of steps, the total distance covered, the daily average and the weekly average. 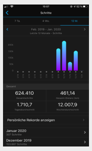	App - Daily feedback on steps, distance covered, time spent on physical activity, calories burned and information about inactivity. 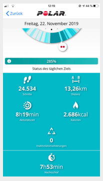	Wearable - Weekly physical activity report. 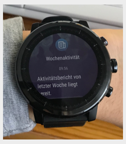	Wearable - Weekly physical activity report. 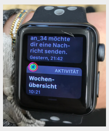
2.3. Self-monitoring of behaviour	App - Daily record of calories burned, steps taken, floors climbed, distance covered, and number of minutes spent on physical activity. 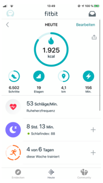	App - Daily record of calories burned, steps taken, floors climbed and distance traveled. 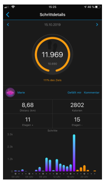	App - Overview of the recorded workouts (strength training, cycling) with information about their intensity. 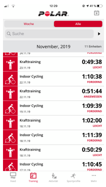	App - Daily record of steps taken, minutes of physical activity, distance traveled and calories burned. 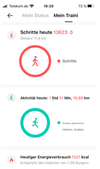	App - Example of physical activity recorded by the wearable including information about the total time of the activity, the distance covered, the calories burned (active calories and total calories), the average heart rate and the average speed. 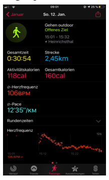
2.6. Biofeedback	App - Feedback on heart rate and average heart rate during a workout, 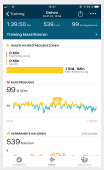	App - Feedback on the current heart rate. 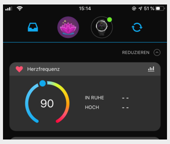	App - Feedback on average, minimum and maximum heart rate during a workout. 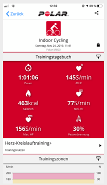	App - Heart rate feedback during a workout. 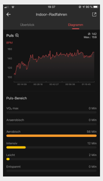	Wearable - Feedback on the current heart rate. 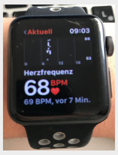

3.1. Social support (unspecified)	App - The left picture shows the possibility to add friends and the right picture shows the possibility to join different groups in order to interact with other users. 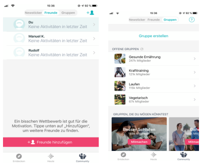	App - The left picture shows the possibility to add friends and the right picture shows the possibility to join different groups in order to interact with other users. 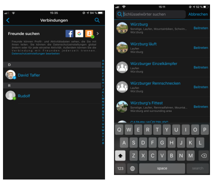	Web-App - The picture shows the possibility to join different groups in order to interact with other users. 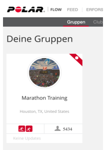	App - The image shows the ability to add friends to share activities. 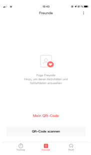	App - The image shows the ability to add friends to share activities. 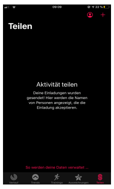
3.3. Social support (emotional)	App - The picture shows messages in the app. In this case, the messages inform that the user was cheered on by a friend who was friendly but also ridiculed. 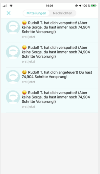	App - This is a notification informing the user that another user has commented on his activity. 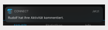	App - This is a notification informing the user that another user has commented on and liked his activity. 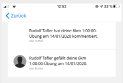	Due to technical problems - receipt and confirmation of a friendship request - no statement could be made for this BCT and it was coded as "not available".	/
4.1. Instruction on how to perform the behavior	App - Overview of training sessions that differ in their duration and the calories burned. 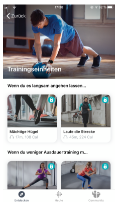	App - The possibility to search for existing workouts with step-by-step instructions or to create workouts. 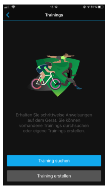	/	/	/
5.1. Information about health consequences	Web-App - Detailed information regarding the recommended 10,000 steps and 30 minutes of physical activity per day and their effects on overall health. 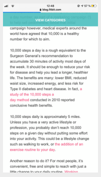	App - Information that the continuous achievement of the activity goals can have a positive effect on heart health, the stability of the bones, the mind and sleep. 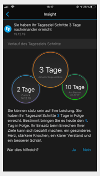	Web-App - Information that the level of physical activity has a positive impact on health and in this case can lead to a longer life expectancy. 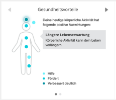	Wearable - Information about the effect that a certain physical activity has on the user's fitness. In this case, the user is informed that the corresponding physical activity has no benefits for his fitness. 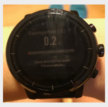	/
5.4. Monitoring of emotional consequences	/	/	App - The user is given the opportunity to indicate his emotional state during the workout by selecting one of 5 smileys that correspond to different moods. 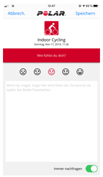	/	/
6.1. Demonstration of the behavior	App - The user has the option of being shown certain exercises through video sequences. 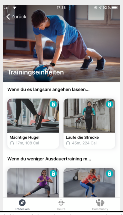	App - The user has the option of being shown certain exercises through video sequences. 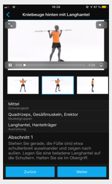	/	/	/
6.2. Social comparison	App - By adding friends, the user is able to compare himself with them. 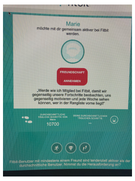	App - By adding friends, the user is able to compare himself with them. 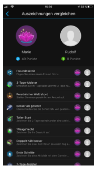	App - It is possible to view precise information about workouts from other users and this enables the user to compare himself with the performance of other users. 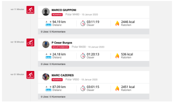	/	App - By adding friends, the user is able to compare himself with them. 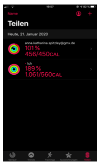

7.1. Prompts/cues	Wearable - Cue to walk 239 steps to achieve the hourly activity goal.<br>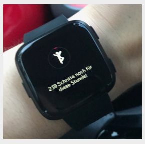	Wearable - Cue that 0 out of 10,100 steps required to achieve the daily step goal have been taken.<br>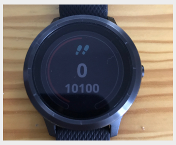	Wearable - Cue that it is time for more exercise and the user is asked to move, stand and walk around.<br>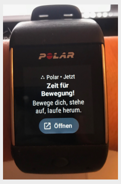	Wearable - Warning that the user is inactive and has been sitting for too long. The user is asked to stand up and move around for a minute.<br>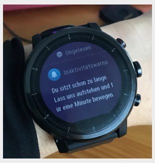	Wearable - Cue informing the user that it is time to stand up and to move around for one minute.<br>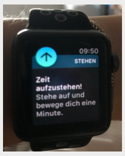
8.1. Behavioral practice/rehear sal	App - The opportunity to practice and improve certain exercises that are demonstrated in video sequences.<br>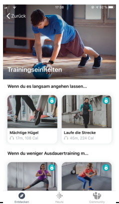	App - The opportunity to practice and improve certain exercises that are demonstrated in video sequences.<br>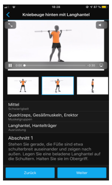	/	/	/
8.7. Graded tasks	App - List of different tasks in which the user has to complete a certain number of steps in one day in order to receive a badge.<br>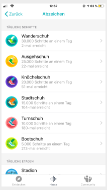	App - List of different tasks in which the user has to complete a certain number of steps in one day in order to receive a badge.<br>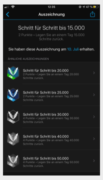	/	/	App - List of different tasks which the user can achieve by repeatedly achieving activity goals in order to receive certain awards.<br>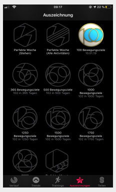
9.1 Credible Source	App - The sources mentioned regarding the guidelines for physical activity are the Surgeon General and a scientific stiry.<br>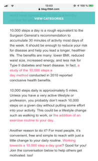	App - The World Health Organization is mentioned here as the credible source.<br>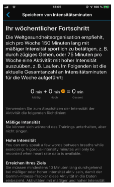	/	App - The World Health Organization is mentioned here as the credible source.<br>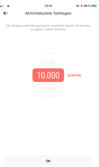	/
10.3. Non-specific reward	Wearable - A colorful hint that the daily activity goal of 10,000 steps has been reached.<br>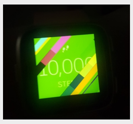	Wearable - A colorful hint that the daily activity goal has been reached.<br>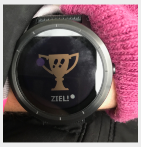	App - On this day, the user is congratulated for the level of physical activity exceeding his daily physical activity goal.<br>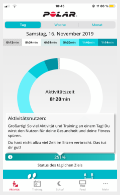	/	App - Here is an award that the user received for his first running training.<br>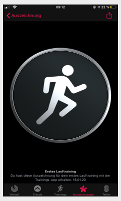
10.4. Social reward	App - The user has the option of sharing recorded physical activities with other users in order to get approval from them.<br>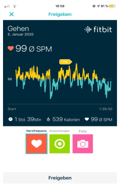	App - The user has the option of sharing recorded physical activities with other users in order to get approval from them.<br>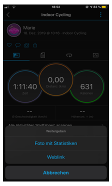	App - The user has the option of sharing recorded physical activities with other users in order to get approval from them.<br>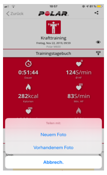	App - The user has the option of sharing recorded physical activities with other users in order to get approval from them.<br>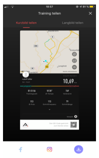	App - The user has the option of sharing recorded physical activities with other users in order to get approval from them.<br>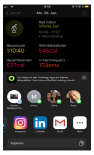
10.6. Non-specific incentive	App - The user is informed that he receives trophies for achieving certain goals and tasks.<br>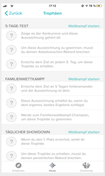	App - The user is informed that he receives trophies for achieving certain goals and tasks.<br>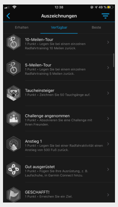	/	/	App - The user is informed that he receives trophies for achieving certain goals and tasks.<br>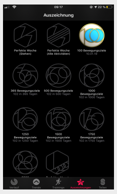

12.5. Adding objects to the environment	The Wearable	The Wearable	The Wearable	The Wearable	The Wearable
14.4. Reward approximation	App - Depending on how close the user gets to his goal of walking 250 steps per hour a total of 9 out of 9 hours a day, he receives a more positive feedback. 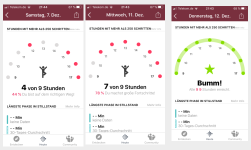	/	/	/	/
14.6. Situation-specific reward	App - The wearable rewards the user with positive feedback when he reaches his goal of 250 steps per hour after being promted to be more physically active by the wearable. 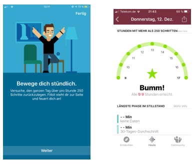	App -The user receives an award if he records an activity on the Chinese New Year. 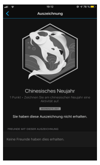	/	/	App - The user receives an award if he achieves all activity goals in January 2020 for 7 consecutive days. 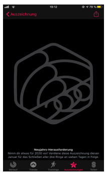
15.1. Verbal persuasion about capability	/	App - The wearable assures the user that he can achieve his daily step goal, which he has now reached three days in a row, for 4 days in a row. 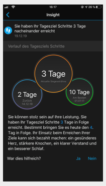	/	/	App - The message from the wearable to the user motivates the user and states that the user can achieve his physical activity goal, despite the fact that he has moved little the previous day. 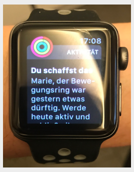
15.3. Focus on past success	Web-App - The user receives information about the badges and trophies for physical activities that he has received in the past. 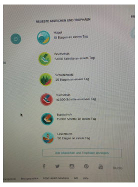	App - The user is informed that he has reached his daily step goal three days in a row. 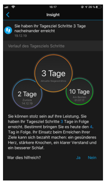	/	App - The user will be informed on which days he was sufficiently physically active from January 13th to 19th. 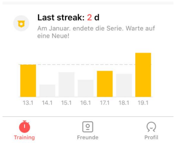	Wearable - The user is informed in an overview of the past week how often he has exceeded his calorie goal. 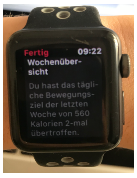
